# Biodegradable metals for bone fracture repair in animal models: a systematic review

**DOI:** 10.1093/rb/rbaa047

**Published:** 2020-12-03

**Authors:** Jiazhen Zhang, Zhizhong Shang, Yanbiao Jiang, Kui Zhang, Xinggang Li, Minglong Ma, Yongjun Li, Bin Ma

**Affiliations:** 1 State Key Laboratory of Nonferrous Metals and Process, GRINM Group Corporation Limited (GRINM), No. 2, XinJieKouWai St., HaiDian District, Beijing 100088, P.R. China; 2 GRIMAT Engineering Institute Co., Ltd, No. 11, Xingke East St., Yanqi Economic Development Zone, Huairou District, Beijing 101407, P.R. China; 3 General Research Institute for Nonferrous Metals, No. 2, XinJieKouWai St., HaiDian District, Beijing 100088, P.R. China; 4 School of Basic Medical Sciences, Evidence-Based Medicine Center, Lanzhou University, No 199, Donggang West Road, Chengguan District, Lanzhou 730000, P. R. China

**Keywords:** biodegradable metal, bone fracture, animal model, systematic review, regulatory science, safety and effectiveness

## Abstract

Biodegradable metals hold promises for bone fracture repair. Their clinical translation requires pre-clinical evaluations including animal studies, which demonstrate the safety and performance of such materials prior to clinical trials. This evidence-based study investigates and analyzes the performance of bone fractures repair as well as degradation properties of biodegradable metals in animal models. Data were carefully collected after identification of population, interventions, comparisons, outcomes and study design, as well as inclusion criteria combining biodegradable metals and animal study. Twelve publications on pure Mg, Mg alloys and Zn alloys were finally included and reviewed after extraction from a collected database of 2122 publications. Compared to controls of traditional non-degradable metals or resorbable polymers, biodegradable metals showed mixed or contradictory outcomes of fracture repair and degradation in animal models. Although quantitative meta-analysis cannot be conducted because of the data heterogeneity, this systematic review revealed that the quality of evidence for biodegradable metals to repair bone fractures in animal models is ‘very low’. Recommendations to standardize the animal studies of biodegradable metals were proposed. Evidence-based biomaterials research could help to both identify reliable scientific evidence and ensure future clinical translation of biodegradable metals for bone fracture repair.

## Introduction 

Fracture-related musculoskeletal diseases have become one of the leading causes of disability, and the number of orthopedic fracture patients worldwide is still accruing [1]. Osteoporosis and motor vehicle accidents are the main causes of fractures. There were 56 million patients with fractures due to osteoporosis worldwide in 2006 [2], and the high-risk population for osteoporotic fractures will reach 316 million by 2040 [3]. As many as 2.9 million patients suffer femoral shaft fractures each year from traffic accidents alone [4]. In addition, the complexity of fracture sites (head, spine, limbs, etc. [5]), injury mechanisms (transverse fractures, oblique fractures, comminuted fractures, etc. [6, 7]) and fracture types [8] has turned treatment a thorny clinical issue.

At present, internal fixation devices are mainly used to fix fractures in clinical treatment [9]. The commonly used materials for internal fixation devices include metals, such as titanium and its alloys, stainless steel and cobalt chromium alloys [10], and biodegradable polymers and their composites [11]. Conventional metal-based implants have become mainstreamed in clinical practice due to their excellent mechanical properties and long history of clinical use. However, the mismatch of the modulus between implants and bones can easily lead to bone resorption from stress shielding, which can further affect the quality of bone formation and the stability and durability of implants [12, 13]. Besides, secondary surgery may need to remove the traditional metal-based internal fixation device, increasing the patient’s pain and financial burden [14, 15]. Other safety risks may include toxic side effects and local tissue reactions caused by the long-term retention of metal particles and ions from device degradation and corrosion in the body [12, 16, 17].

**Figure 1:**
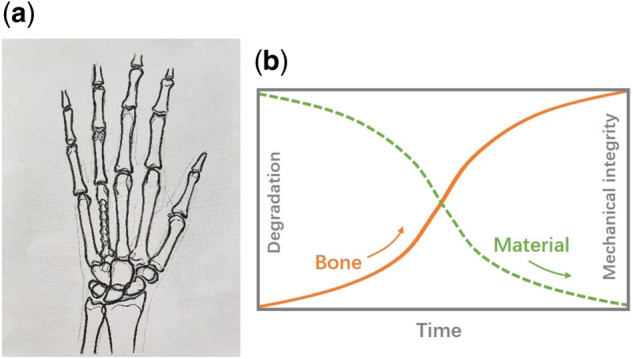
Schematic diagram of biodegradable metals for bone fracture repair. (a) Fixation of the metacarpal fracture with a biodegradable metal implant, and (b) illustration of the unique combinatorial properties of both mechanical integrity and material degradation [23–25]

Biodegradable metals represented by magnesium, zinc and their alloys have been proposed as internal fixation materials for fractures with great potentials [18]. This novel class of metallic material has obvious advantages. First, their mechanical properties support strong fixation for early fracture healing by providing effective fixation strength during the initial implantation [19]. Second, the implant will gradually degrade in the physiological environment, avoiding the injury caused by traditional metallic fixation devices and bone loss from stress shielding [20, 21]. Related studies have also shown [22] that ions produced by material degradation can stimulate the regeneration of bone tissue around the implant. Such biodegradable internal fixation devices used for fracture repair should have strength and degradation rate that match the fracture healing cycle. An ideal biodegradable implant is demonstrated in Fig. 1 [23–25].

However, previous studies have also shown [26–28] that magnesium exhibit high corrosion sensitivity and non-uniform corrosion behavior under stress or in simulated physiological environments rich in chloride ions. There may also be significant reduction of the mechanical properties after implantation and the risk of losing fixation or support functions before complete healing of fracture. At the same time, excessive ions and gases released from rapid degradation may cause over-accumulation of metal ions in the local tissue, metabolism overload and air cavity formation around the implant [29, 30].

*In vitro* simulated experiments of different alloys and products, given the effects of alloying element on mechanical and corrosion properties, residual mechanical stress, coatings and experimental conditions such as buffer systems and inorganic ions, are very challenging in predicting *in vivo* performance [31]. Hence, animal studies are indispensable for evaluating the safety and performance of such materials and implants.

Animal studies of biodegradable metal implants could provide relevant data for preclinical evaluation of such products and lay a preliminary foundation for their future clinical research. Such studies have investigated the biocompatibility, *in vivo* degradation, osteogenesis and fracture repair of biodegradable metal implants. However, these studies have the following deficiencies, over-simplification of animal model construction, a narrow focus on biocompatibility evaluation, and evaluating the effect of bone repair with defects size below the critical size. Ideal animal studies on biodegradable metal devices for internal fixation should be guided by expected clinical indications and establish animal models to evaluate safety and performance of implants and materials [29, 32]. Because the degradation rate and tissue response of implants in different animal models of fractures are different [33], the validity and comparability across different animal studies are very important. Such studies bear implications for subsequent clinical trials. However, the diversity of material systems, model construction and evaluation methods used in current animal studies for biodegradable metals have made it difficult to assess the validity and comparability across different studies, and have also led to apparently conflicting research results [34–37]. Therefore, a systematic approach is needed to analyze the current animal studies on biodegradable metals in search of evidence for their potential clinical translation [38].

Systematic reviews (SRs), as key methods to conduct research on evidence-based medicine, have been frequently used to quantitatively and/or qualitatively review clinical-related studies [39]. Evidence-based research methods such as SRs have also been used to investigate pre-clinical studies [40]. Compared to traditional literature reviews which are heavily subject to authors’ professional ability without following quality standards and protocols, SRs not only summarize and recommend convinced evidence of pre-clinical research topics, but also give suggestions and evidence leading to related clinical studies [41, 42]. Among many kinds of pre-clinical studies, animal studies are critical to evaluate safety and performance of biomaterials and related medical devices [43]. Furthermore, evidence-based research with SRs is new and rarely reported to the biomaterials field in terms of pre-clinical animal studies [44, 45]. In addition, SRs of animal studies have the potential to reduce the challenges during the translation of animal data to clinical trials, which could improve the efficiency to demonstrate safety and efficacy of medical products [46].

This study is a SR of published animal studies of biodegradable metals versus traditional materials (i.e. non-degradable metals and absorbable polymers) for fracture repair. It provides a comprehensive analysis of the material composition, structure, implant design, animal model, anatomical site, fracture, follow-up time, fracture healing and degradation properties of the relevant materials and implants. The safety and effectiveness of such biodegradable metals for fracture repair are explored. The feasibility, benefits and risks of clinical translation and subsequent clinical trials are also evaluated. To the best of our knowledge, this study is the first SR on biodegradable metals focusing on their ability to repair bone fractures in animal models.

## Materials and methods

### Purpose of study

This study intends to conduct a SR on animal studies of biodegradable metals for the repair of bone fractures and defects. As a result, this study adopts a search strategy and literature screening process that include the comprehensive results on biodegradable metals used for repairing bone fractures and defects. However, due to the differences in the causes, mechanisms, healing and treatment principles of bone fractures and defects, this study only systematically reviews evidence relevant to fracture repair, while those on bone defects will be reported separately.

### Data inclusion/exclusion criteria

#### Participants

Studies that include animal models of bone fractures, with no limit on the animal species nor fracture modeling methods.

#### Interventions

Degradable metals and their alloys, modified degradable metals and their alloys (composites, coating modification and surface modification).

#### Comparisons

① Non-degradable metals, such as titanium, titanium alloy, stainless steel and cobalt chromium alloys; ② absorbable polymers, such as polylactic acid (PLA) and ③ other materials, such as calcium phosphate ceramic, autogenous bone, allogeneic bone and absorbable or degradable composites for traditional clinical applications (e.g. ceramics).

#### Outcome measures

##### Outcome measures for fracture healing

Primary outcome measures include ① new bone formation: Increased density shadows (of trabecular bone, epiphysis, etc.) are detected inside or around the fracture line by imaging methods; ② fracture healing: Fracture line gradually disappears in the observation by imaging methods; ③ bone volume: Micro-CT scans are used to analyze bone tissue reconstruction and quantify bone volume and ④ total callus volume: Micro-CT scans are used to quantify the total volume of the callus formation. Secondary measures include ① maximum stress: a three-point or four-point bending test is performed on the specimen to obtain biomechanical properties.

##### Implant-related degradation outcome measures

① Hydrogen generation: Observation of gas shadows by imaging; ② implant degradation: observation of rupture or corrosion of implants at fracture sites by imaging; ③ remaining volume of implants: quantitative measure of the remaining volume of the implant by Micro-CT.

Given the different species of animals in the included studies, e.g. rats [12, 36, 47–50], rabbits [51], dogs [35, 37, 52], sheep [34], pigs [53], there must be differences in the fracture healing time. To facilitate the combinatorial analysis of the outcome measures, we divided the whole follow-up process of the included studies into four measurement periods, which are T1, the initial period (0 <T1 ≤ 1/4T); T2, the mid-term period (1/4 T <T2 ≤ 2/4T); T3, the long-term period (2/4 T <T3 ≤ 3/4T); T4, the terminal period (3/4 T <T4≤T), with ‘T’ representing the whole follow-up time.

#### Study design

Controlled studies were included, with no restriction on whether they are randomly grouped. Self-control studies were excluded to ensure the quality of inclusion and eliminate the effects of degradation products on the body of experimental animals and interference with fracture evaluation [50].

#### Data inclusion and exclusion

This study strictly follows the above population, interventions, comparisons, outcomes and study to extract data after carefully reviewing the title, abstract and full text of each article. Only studies that are comply with the following criteria are included: (i) biodegradable metals as interventions; (ii) animal studies of bone fractures as study objects and (iii) controlled studies.

### Search strategy

We searched the PubMed (1966 to August 2019), Ovid-Embase (1980 to August 2019), The Cochrane Library (1989 to August 2019), Web of science (from inception to August 2019), China National Knowledge Infrastructure or CNKI (from inception to August 2019), China Science Periodical Database or CSPD (from inception to August 2019), Chinese Scientific Journal Database or VIP (from inception to August, 2019), Wanfang Database or Wanfang (from inception to August, 2019) and China Biomedical Literature Database or CBM (from inception to August 2019). Supplementary search included Scopus (from inception to August 2019). In addition, the references of included studies were checked. Authors of studies with incomplete data were contacted to obtain the required information. The retrieval method was a combination of free words and medical subject heading (Mesh). See Supplementary data for Chinese and English search strategies.

### Paper selection and data extraction

Two trained researchers (Z.S. and Y.J.) selected the papers and extracted the data in strict accordance with the inclusion/exclusion criteria, and cross-checked them. In case of disagreement, a third party (J.Z.) would decide. Data were extracted according to the pre-established full-text data extraction checklist, including: ① basic parameters of the included studies: including the species, age, weight, sample size, fracture model, types of interventions and follow-up time of the experimental animals; ② outcome measures: (i) outcome measures for fracture healing: new bone formation, fracture healing, bone volume, total callus volume and maximum stress; (ii) Outcome measures for implant degradation: gas generation, implant degradation and remaining implant volume.

### Risk assessment of bias

Based on SYRCLE's risk of bias tool for animal studies [54], 2 trained researchers (K.Z. and M.M.) independently evaluated and cross-checked the inherent risk of bias in the included studies, covering selection bias, implementation bias, measurement bias, follow-up bias, report bias and other bias from a list of 10 questions or tools. A difference in opinions were negotiated or decided by a third party (B.M.). The answer to the assessment questions (tools) should be either ‘yes’ that indicated low risk of bias, or ‘no’ that indicated high risk of bias. For unclear items an answer with ‘unclear’ was assigned.

### Quality assessment of evidence

Whether the results of SR of animal studies can lead to clinical translation depends on the quality of the evidence. The CERQual tool (Confidence in the Evidence from Reviews of Qualitative research) [55, 56] developed by Cochrane Collaboration for the grading and evaluation of evidence assess the quality of the following four aspects: ① methodological limitations; ② correlation; ③ consistency of results and ④ adequacy of data. To assess the quality of evidence for this SR, we evaluated the above four criteria individually, and then the result of each criterion was combined to calculate a level of evidence of high, moderate, low or very low [56].

## Results

### Systematic search outcomes

**Figure 2:**
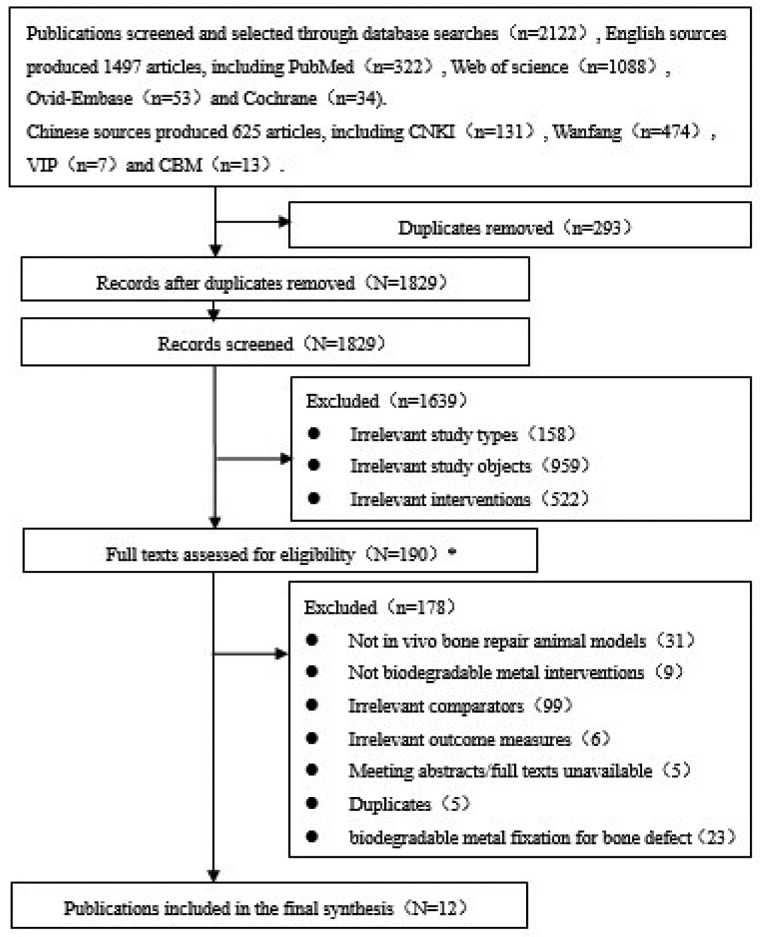
Study screening and selection process

After searching five English databases and four Chinese databases, a total of 2122 relevant articles were yielded in the preliminary searches, of which 625 were in Chinese and 1497 in English. After excluding duplicates, a total of 1829 articles were obtained. After reviewing titles and abstracts, a total of 190 articles were collected after excluding 158 reviews, comments and secondary-study articles that did match types of study, 959 clinical or *in vitro* study articles that did not match objects of study, as well as 522 articles that use calcium phosphate bone cements or bioresorbable polylactides as interventions. and studies that did not meet the inclusion criteria, only 12 animal studies on biodegradable metals for bone fracture repair were finally included, including 8 English [12, 34, 35, 47, 48, 50, 51, 53] and 4 Chinese articles [36, 37, 49, 52]. The screening and selection processes are shown in Fig. 2.

Twelve animal studies on biodegradable metals for bone fracture repair were finally included, including eight English [12, 34, 35, 47, 48, 50, 51, 53] and four Chinese articles [36, 37, 49, 52]. The screening and selection processes are shown in Fig. 2. Animal studies on biodegradable metals for bone fracture repair were conducted by several groups in recent years. The 12 included studies from China, America, Japan and Switzerland were published between 2015 and 2019.

### Summary of included studies

This review included three types of biodegradable metals for bone fracture repair in animal studies, including pure magnesium [48, 50, 51], magnesium alloys [12, 34–36, 47, 49, 52, 53] and zinc alloys [37]. The 12 studies included 8 randomized controlled studies [, 49–53] and 4 controlled studies [12, 34, 47, 48]. The animal species included in the study were rats [12, 36, 47–50], rabbits [51], dogs [35, 37, 52], sheep [34] and pigs [53]; animal age and weight were mostly between 2 months [36, 48] and 24 months [53] and between 200 g [36] and 90 kg [53]. The sample size was between 2 [53] and 60 [12]. Fracture models included femoral condyle fractures [34, 51], femoral fractures [12, 36, 47, 48, 50], tibial fractures [35, 49], superior orbital and patella fractures [53], rib fractures [52] and mandibular fractures [37]. The follow-up duration ranged from 3 weeks [48] to 48 weeks [37]. The detailed information of included studies is shown in Tables 1 and 2.

**Table 1:** The characteristic of the included animal studies

Implant type	Author (year)	Country	Study design	Species	Sample size (T/C)	Age	Body weight	Model	Follow-up time
Mg	Han et al. (2015) [51]	China	Randomized	Rabbits/New Zealand White	24/12	Skeletally mature	3.0 ± 0.5 kg	Femoral intercondylar fracture	24 weeks
	Li et al. (2019) [48]	China	Controlled	Rats/SD	16/16	8 weeks	250–300 g	Femoral shaft fracture	3 weeks
	Zhang et al. (2016) [50]	China	Randomized	Rats/SD	26/23	6 months		Femoral shaft fracture	12 weeks
Magnesium alloy	Chou et al. (2019) [47]	USA	Controlled	Rats/SD	15/15		250–300 g	Right hind limb femur fracture	14 weeks
	Kong et al. (2018) [34]	China	Controlled	Goats	12/12	Mature		Femoral condyle fracture	6 months
	Kong et al. (2018) [34]	China	Controlled	Goats	12/12	Mature		Femoral condyle fracture	6 months
	Marukawa et al. (2016) [35]	Japan	Randomized	Dogs/beagle	3/3	1 year	∼10 kg	Crank-shaped tibial fracture	12 weeks
	Marukawa et al. (2016) [35]	Japan	Randomized	Dogs/beagle	3/3	1 year	∼10kg	Crank-shaped tibial fracture	12 weeks
	Schaller et al. (2018) [53]	Switzerland	Randomized	Pigs/Yucatan miniature	2/2	24 months	80 ± 10 kg	Upper iliac crest and humerus osteotomy	9 months
	Ma (2015) [36]	China	Randomized	Rats/SD	20/20	8 weeks	224 ± 24 g	Femoral shaft fracture	12 weeks
	Min et al. (2015) [52]	China	Randomized	Dogs/beagle	10/5	12 months	>12 kg	Rib fractures	12 weeks
	Wang (2016) [49]	China	Randomized	Rats/SD	3/3		250–400 g	Fracture of tibia	8 weeks
	Wang (2016) [49]	China	Randomized	Rats/SD	3/3		250–400 g	Fracture of tibia	8 weeks
	Li et al. (2018) [12]	China	Controlled	Rats/SD	Total: 60	6 months		Femoral shaft fracture	12 weeks
	Li et al. (2018) [12]	China	Controlled	Rats/SD	Total: 60	6 months		Femoral shaft fracture	12 weeks
Zn alloy	Wang (2018) [37]	China	Randomized	Dogs/beagle	12/12	10–12 months	10–15 kg	Mandible fracture	12 months

**Table 2:** Materials and implants of the included animal studies

Implant type	Author (year)	Control implant	Degradable metal implant components	Coating	Implant shape		Implant specifications	
					T	C	T	C
Mg	Han et al (2015) [51]	PLLA	(99.99 wt.% Mg; 0.002 wt.% Si; 0.0015 wt.% Fe; 0.0008 wt.% Al; 0.0008 wt.% Mn; 0.0002 wt.% Ni; 0.0003 wt.% Cu		Screw	Screw	Major diameter: 2.7 mm; core diameter: 2.1 mm; pitch: 1 mm; length: 27 mm	Major diameter: 2.7 mm; core diameter: 2.1 mm; pitch: 1 mm; length: 27 mm
	Li et al (2019) [48]	Stainless steel	Mg		Pin	Pin	Diameter: 1.5 mm, length: 25 mm	Diameter: 1.5 mm, length: 25 mm
	Zhang (2016) [50]	Stainless steel	(99.99%) magnesium		Needle	Needle	Outer diameter: 1.27 mm; inner diameter: 0.9 mm	Outer diameter: 1.27 mm; inner diameter :0.9 mm
Magnesium alloy	Chou et al. (2019) [47]	Ti6Al4V	Mg-4.0%Y-2.0% Zn-1.0%Zr-0.6%Ca in wt. %		Nail	Nail	Diameter: 1.66 mm × ; length: 15 mm	Diameter: 1.66 mm × ; length: 15 mm
	Kong et al. (2018) [34]	PLA	Mg–Nd–Zn–Zr		Screw	Screw	Outer diameter: 4.5 mm ; length: 45 mm	Outer diameter: 4.5 mm ; length: 45 mm
	Kong et al. (2018) [34]	PLA	Mg–Nd–Zn–Zr	Brushite coating	Screw	Screw	Outer diameter: 4.5 mm ; length: 45 mm	Outer diameter: 4.5 mm ; length: 45 mm
	Marukawa et al. (2016) [35]	PLLA	Mg–Y–Zr		Screw	Screw	Shaft diameter: 2.6 mm; full length: 13 mm	Shaft diameter: 2.6 mm; full length: 13 mm
	Marukawa et al. (2016) [35]	PLLA	Mg–Y–Zr	Ca+P+O_2_ of oxide film	Screw	Screw	Shaft diameter: 2.6 mm; full length: 13 mm	Shaft diameter: 2.6 mm; full length: 13 mm
	Schaller et al. (2018) [53]	PLGA	WE43 alloy	AHC ^®^ plasma electrolytic coating	Plate; screw	Plate; screw	Plate (Φ5.1 x 14.1 x 0.6 mm; Φ5.1 x 23.1 x 0.8 mm) Screw(Φ1.5 × 4-6mm)	Plate (Φ5.1 × 14.1 × 0.6 mm; Φ5.1 × 23.1 × 0.8 mm) Screw(Φ1.5 × 4-6mm)
	Ma (2015) [36]	Pure iron	Mg (99.93%), Mn (0.017%), Si (0.021%), Al (0.014%), Ca (0.0033%), Fe (0.0032%), Cu (0.0018%), Ni (0.0009%)		Screw	Screw	Diameter : 1 mm	Diameter: 1 mm
	Min et al. (2015) [52]	Steel	Zn（5.6210%）, Fe（0.0038%）, Si（0.0016）, Ni（0.0005%）, Cu（0.0005%）, Al（0.0085%）, Mn（0.0004%）, Mg（balance）		Rib plate	Rib plant	Length: 35 mm ;height: 8 mm; width: 12 mm	Length: 35 mm ;height: 8 mm ;width: 12 mm
	Wang (2016) [49]	Ti alloy	Mg–Nd–Zn–Zr		screw	screw	Diameter: 1.5 mm ; length: 20 mm	Diameter: 1.5 mm ; length: 20 mm
	Wang (2016) [49]	Ti alloy	Mg–Nd–Zn–Zr	Hydroxyapatite /HA	Screw	Screw	Diameter: 1.5 mm ; length: 20 mm	Diameter: 1.5 mm ; length: 20 mm
	Li et al. (2018) [12]	Stainless steel	Mg–Nd–Zn–Zr	PLA/brushite (CaP)+ZA	Nail	Nail	Diameter: 1.5 mm; length: 80 mm	Diameter: 1.5 mm; length: 80 mm
	Li et al. (2018) [12]	Stainless steel	Mg–Nd–Zn–Zr	PLA/brushite (CaP)	Nail	Nail	Diameter: 1.5 mm; length: 80 mm	Diameter: 1.5 mm; length: 80 mm
Zn alloy	Wang (2018) [37]	PLLA	Zn alloy （chemical composition not mention）		Plate and screw	Plate and screw	Plate (thickness 1 mm) screw (diameter 2 mm, length 7 mm)	Plate (thickness 1 mm), screw (diameter 2 mm, length 7 mm

The outcome measures included in each study report and the measurement time points were different. There was also a large difference in the measuring methods and judgment criteria of the outcome measures.

Five measures of fracture healing were included. ① New bone formation: this measure was reported in eight studies [12, 34, 36, 49, 51–53], which used the experimental animals of rabbits [51], goats [34], pigs [53], rats [12, 36, 49, 50] and dogs [52]. The components of the implants were magnesium [50, 51] and magnesium alloys [12, 34, 36, 49, 52, 53], and the measurement time points were between 2 months [49] and 9 months [53]. X-ray and microscopic observations [12, 50, 51], CT [34, 49, 52, 53], histological analysis [36, 49, 50] and other methods were used to measure new bone formation. ② Fracture healing: this measure was reported in seven studies [35–37, 48, 49, 52, 53], which used the experimental animals of rats [36, 48, 49], dogs [35, 37] and pigs [53]. The compositions of the implants were magnesium [48], magnesium alloys [35, 36, 49, 52, 53] and zinc alloys [37], with measurement time points ranging from 3 weeks [48] to 12 months [37]. Histological analysis [35, 52, 53], morphological observation [49], X-ray [36], CT [37] and other methods were used to measure fracture healing. ③ Bone volume: this measure was reported in four studies [12, 36, 37, 50], which used the experimental animals of rats [12, 36, 50] and dogs [37]. There were large differences in implant composition (magnesium alloys [12, 36], pure magnesium [50] and zinc alloys [37]), and the measurement points were also different (12 weeks [12, 36, 50] and 12 months [37]). ④ Maximum stress: this measure was reported in five studies [12, 36, 37, 50, 51]. Although the maximum stress of the new bone was all measured by mechanical testing equipment, the experimental animals (rabbit [51], rat [12, 36, 50] and dogs [37]), implant components (pure magnesium [50, 51], magnesium alloys [12, 36] and zinc alloys [37]) and measurement points were all different (12 weeks [12, 36, 50], 24 weeks [51] and 12 months [37]). ⑤ Total callus volume: only one study [36] reported this measure.

Three implant degradation measures were included. ⑥ Remaining implant volume: this measure was reported in four studies [37, 47, 51, 53], in which the used experimental animals (rabbit [51], mouse [47], pig [53] and Dog [37]), implant compositions (pure magnesium [51], magnesium alloy [47, 53] and zinc alloy [37]) and measurement points (12 weeks [47], 24 weeks [51], 9 months [53] and 12 months [37]) were different. There were also divergences in the measurement methods of the remaining volume of the implant (including CT [37, 47, 51] and histological analysis [53]). ⑦ Implant degradation: although the five studies [34, 35, 44, 49] including this measure all measured the degradation of the implants by CT, the experimental animals (rats [47–49], goats [34] and dogs [35]), implant components (pure magnesium [48] and magnesium alloy [34, 35, 47, 49]) and measurement points (3 weeks [48], 8 weeks [49], 12 weeks [35], 14 weeks [47] and 6 months [34]) were different. ⑧ Hydrogen generation: this measure was reported in five studies [34, 47, 49, 52, 53]. There were differences in the experimental animals used in each study (rat [47, 49], goat [34], pig [53] and dog [52]), measurement points (8 weeks [49] to 9 months [53]) and measurement methods (general morphological observations [52], X-rays [49] and CT [34, 47, 53]).

Therefore, the heterogeneity between studies was difficult to eliminate in this review, which means that we could not perform a meta-analysis on the data available. Only a descriptive analysis was possible.

Based on the inclusion criteria, some of the highly-cited research articles on biodegradable metals were excluded, due to non-fracture models [57–60] and self-control [58] of those animal studies.

In the same study: ‘a’: the biodegradable metals group without coatings; ‘b’: the biodegradable metals group with coatings. In the study [12], the samples of the two sub-groups (a, b) were both biodegradable metals with coatings.

The follow-up process of the included studies is divided into four periods, which are the initial period, the mid-term period, the long-term period, the terminal period.

### Repair of fractures with pure magnesium materials

Only three included studies [48, 50, 51] explored the repair of fractures with pure magnesium materials, using 36 [51], 32 [48] and 49 [50] animals, respectively. The animal species used were New Zealand White Rabbits [51] and Sprague-Dawley rats [48, 50]. The animal ages are skeletal maturity [51], 8 weeks old [48] and 6 months old [50], weighing 3.0 ± 0.5 kg [51] and 250–300 g [48]. One study [50] did not report the weight of experimental animals. The follow-up durations were 24 weeks [51], 3 weeks [48] and 12 weeks [50]. The fracture models used were femoral condyle fractures [51] and femoral fractures [48, 50]. The implants for study [51] were high-purity magnesium (99.99 wt.%) and PLLA screws (2.7 mm in major diameter, 2.1 mm in core diameter, 1 mm in pitch and 27 mm in length). The implants for study [48] were Mg and stainless-steel machined cylindrical pins (1.5 mm in diameter and 25 mm in length). The implants for study [50] were Mg-IMN (99.99 wt.% Mg) and IMN (stainless steel material, 1.27 mm in outer diameter and 0.9 mm in inner diameter).

Study [51] compared the effects of pure magnesium and the absorbable polymer PLLA on fracture repair. The results were as follows. ① new bone formation: throughout the fracture healing process, the density and quality of new bone and trabeculae in the pure magnesium group were better than the control group; ② maximum stress: during the fracture healing process, the strength of bone in the pure magnesium group gradually decreased, but the study did not report the bone strength of the control group; ③ the remaining volume of the implant: with the extension of implantation time, the remaining volume of the implant of the pure magnesium group gradually decreased. The remaining volume of the implant in the control group was not reported.

**Figure 3:**
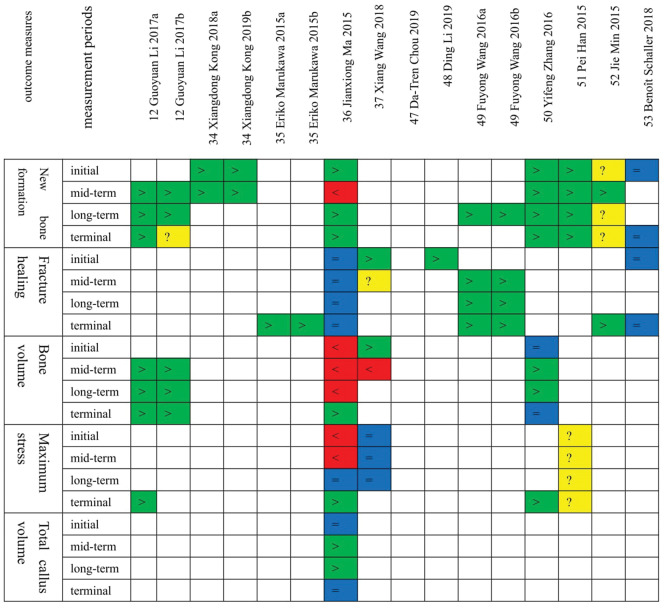
The outcome measures of new bone formation

**Figure 4:**
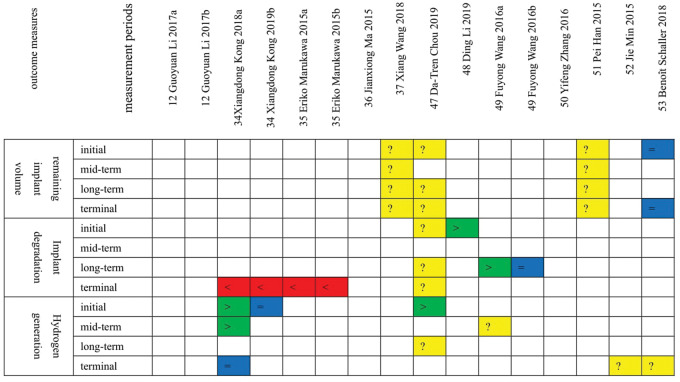
The outcome measures of implant degradation. ‘>’, green: the effect of the biodegradable metals group is superior to the control group; ‘<’, red: the effect of the control group is superior to the biodegradable metals group; ‘=’, blue: there is no difference between the biodegradable metals group and the control group; ‘?’, yellow: there is no comparison between the biodegradable metals group and the control groups, or only outcomes of the biodegradable metals group were reported; ‘/’, blank: There is no outcome for this period

Both studies [48, 50] compared the effects of pure magnesium and non-degradable stainless steel on fracture repair. The results of study [48] showed that pure magnesium was better than the control group in osteoblast viability and osteogenesis, and the implant of the pure magnesium group was significantly degraded. The results of study [50] were as follows. New bone formation: During the entire fracture healing process, the rate and volume of new bone callus formation in the pure magnesium group were greater than those in the stainless-steel group, and at the end of fracture healing, the pure magnesium group had formed mature lamellar bone. Bone volume: there was no statistically significant difference in bone volume between the two groups at the initial and final stages of fracture healing. During the mid- and long-term fracture healing, the bone volume of the pure magnesium group was greater than that of the stainless-steel group. The difference in bone volume between the groups was statistically significant. Maximum stress: the four-point bending biomechanical test at the end of fracture healing showed that the maximum compression load of the femoral shaft in the pure magnesium group was greater than that of the stainless-steel group (see Figs 3 and 4).

### Repair of fractures with magnesium alloy materials

A total of eight included studies [12, 34–36, 47, 49, 52, 53] explored the repair of fractures with magnesium alloy materials, of which five studies [12, 36, 47, 49, 52] compared the repair of fractures with magnesium alloys and non-degradable metals and three studies [34, 35, 53] compared the effects of magnesium alloys and degradable polymer materials on fracture repair. The animal sample sizes were between 4 [53] and 60 [12]. The animal species included Sprague-Dawley rats [12, 36, 47, 49], goats [34], beagle dogs [35, 52] and Yucatan miniature pigs [53]. The animal subjects were mostly between 8 weeks [36] and 24 months [53], with body weight between 200 g [36] and 90 kg [53], and follow-up durations between 8 weeks [49] and 9 months [53]. Fracture models included femoral fractures [12, 36, 47], femoral condyle fractures [34], tibial fractures [35, 49], superior orbital and patella fractures [53] and rib fractures [52]. The implants of the control groups included PLA [34], PLLA [35], PLGA [53], iron [36], stainless steel [12, 52] and titanium alloy [47, 49]. The lengths of the implants were between 13 mm [35] and 80 mm [12] and the diameters between 1 mm [36] −4.5 mm [34]. The shapes of implants were mostly screws and nails [12, 34–36, 47, 49, 53], and only study [52] used irregular fixation plates.

#### *Magnesium alloys vs non-degradable metals [* 12, 36, 47, 49, 52*] (see Figs 3 and 4)*.

##### New bone formation

Four studies [12, 36, 49, 52] reported new bone formation. The results were as follows. In the early stage of new bone formation, the density and quality of the new bone in the magnesium alloy group of the study [36] was better than that of the control group. Study [52] showed that the fracture ends of both groups were well aligned and the fracture lines were clear, although no statistical comparisons were conducted between groups. In the middle stage of new bone formation, the quantity and density of newly formed trabeculae and callus of the magnesium alloy group studied [12, 52] were better than those of the control group, while the results of study [36] exhibited just the opposite, with loose trabecula shown in the magnesium alloy group, and the new bone formation worse than that in the control group. In the long term of new bone formation, the quantity and volume of newly formed trabeculae and callus in the magnesium alloy group in studies [12, 36, 49] were better than those of the control group. Study [52] showed that the fracture lines at both ends were blurred and the quantity of callus increased, but no statistical comparison was made between the groups. During the end of new bone formation, studies [12, 36] showed that thickness and quantity of bone trabeculae in the magnesium alloy group were better than those in the control group. Study [52] showed that the fracture lines disappeared and the fractures healed well in both groups, but no statistical comparison were made between the groups.

##### Fracture healing

Three studies [36, 49, 52] reported fracture healing. The results were as follows. In the middle period of fracture healing, the fracture line of the magnesium alloy group of study [49] was gradually blurred, while that of the control group was still clearly visible. In the long-term fracture healing, the fracture line of the magnesium alloy group of study [49] was blurred or disappeared, while that of the control group was still seen. At the end of fracture healing, the fracture line of the magnesium alloy groups studied in [49, 52] disappeared, and the fracture healing was good. However, although the fracture line of the control group disappeared, the fracture healing was not optimum, with some bone trabeculae being irregularly arranged. Study [36] explored the fracture healing effect through the fracture healing score. The results showed that the fracture healing score of the two groups gradually increased over time and the fracture gradually healed during the whole follow-up period. However, there was no statistical difference in the fracture healing scores between the two groups.

##### Bone volume

Only two studies [12, 36] reported bone volume. The results of study [36] showed that the bone volume of the magnesium alloy group was smaller than that of the pure iron group at the early, middle and long-term stages of fracture healing, and the bone volume of the magnesium alloy group was bigger than that of the pure iron group at the end of fracture healing. The results of study [12] showed that the bone volume of the magnesium alloy group was bigger than that of the stainless-steel group throughout the fracture healing process.

##### Maximum stress

Only two studies [12, 36] reported maximum stress. The results of study [36] showed that the maximum stress in the magnesium alloy group was worse than that of the pure iron group in the early and middle stages of fracture healing. During the long-term fracture healing, the maximum stress of both groups increased gradually. However, there was no statistical difference between the groups. At the end of fracture healing, the maximum stress in the magnesium alloy group was higher than that in the pure iron group. The three-point bending test at the end of fracture healing of study [12] showed that the maximum failure load of the femoral shaft of the magnesium alloy group increased, three times that of the stainless-steel group.

##### Total bone callus volume

Only study [36] reported total bone callus volume. The results showed that the total volume of callus in the two groups peaked in the middle period, and then declined. There was no statistical difference between the two groups at the initial and terminal stages of fracture healing. The total volume of callus is larger in the biodegradable metal group than the pure iron group during the mid- and long-term periods.

##### Remaining implant volume

One study [47] reported the remaining implant volume. Since the control group was a non-degradable metal, the study only reported the remaining volume of the implant of the magnesium alloy group, showing that as the fracture healed, the remaining volume of the implant in the magnesium alloy group gradually decreased.

##### Implant degradation

Two studies [47, 49] reported implant degradation. The results were as follows. In the long-term fracture healing, the uncoated magnesium alloy implant in study [49] degraded too quickly, leading to fracture of the intramedullary nail after rat activity, and the fracture did not heal. In the control group, the intramedullary nail maintained good shape with clear boundaries and no degradation traces were visible. The coated magnesium alloy implants and the control group in study [49] maintained relatively complete shape, with clear boundaries, and no visible degradation traces. There was no significant difference in the degradation between the two groups. At the end of fracture healing, study [47] only reported the degradation of the implant in the magnesium alloy group since the control group was a non-degradable Ti6Al4V alloy. The degradation rate of the experimental group was the fastest for the initial stage of implantation, followed by the terminal period, and the slowest in the long-term period.

##### Hydrogen generation

Three studies [47, 49, 52] reported hydrogen generation. The results were summarized as follows. In the early stage of fracture healing, the hydrogen generation of the magnesium alloy group in study [47] was more than that of the control group; In the middle stage of the fracture healing, the uncoated magnesium alloy group in study [49] saw subcutaneous emphysema due to early hydrogen generation; In the long-term fracture healing, the magnesium alloy group of study [47] showed signs of hydrogen generation. At the end of fracture healing, one experimental animal in the magnesium alloy group of study [52] developed subcutaneous gas accumulation.

#### *Magnesium alloy vs. degradable polymers [* 34, 35, 53] (see Figs 3 and 4).

##### New bone formation

Two studies [34, 53] reported new bone formation. The results were as follows. In the early stage of new bone formation, the density and quality of new bone in the magnesium alloy group of study [34] was better than that of the control group. Study [53] showed that there were bone trabeculae and new blood vessels around the fracture lines in both groups. However, there was no statistical difference in the quantitative analysis of new bone formation between the two groups. In the middle stage of new bone formation, the quantity and density of newly formed trabeculae in the magnesium alloy group of study [34] were better than that of the control group; Study [53] showed that more trabeculae were seen around the fracture line in both groups, with promising new bone formation. However, the new bone formation in the two groups was not statistically different.

##### Fracture healing

Two studies [35, 53] reported fracture healing. The results were as follows. In the early stage of fracture healing, both groups of study [53] showed blurred fracture lines and signs of fracture healing. However, no group effect was found in statistical comparison of fracture healing. At the end of fracture healing, the fracture lines of the magnesium alloy group and the control group of study [35] both disappeared. However, while the fracture healing was fine in the experimental group, it was not the case for the control group, with some irregularity in trabeculae. The fracture lines of the experimental animals of the two groups in Study [53] disappeared in this terminal period, and there was no statistical difference in fracture healing between the two groups.

##### Remaining implant volume

One study [53] reported the remaining implant volume. The results showed that there was no statistical difference in the remaining volume of the implant between the initial and final stages of fracture healing. However, compared with the initial stage of fracture healing, the remaining implant volume of the magnesium alloy group increased slightly at the end of fracture healing (volume growth due to new bone formation around the implant), while the volume of the implant in the control group decreased in this period.

##### Implant degradation

One study [35] reported implant degradation. The results showed that at the end of fracture healing, the implants in the magnesium alloy group did not degrade significantly, while the screws made of polymeric biomaterials in the control group were deformed or broken.

##### Hydrogen generation

Two studies [34, 53] reported hydrogen generation. The results were as follows. In the early stage of fracture healing, hydrogen generation from the uncoated magnesium alloy group in study [34] was more than that of the control group. No hydrogen generation was observed in neither the coated magnesium alloy group nor the control group due to the effective inhibition of implant degradation by the coating. In the middle stage of fracture healing, hydrogen generation from the uncoated magnesium alloy group in study [34] was higher than that of the control group. Hydrogen generation in the long-term healing period was not reported in either study. At the end of fracture healing, the implants of the magnesium alloy group of study [53] showed no significant hydrogen generation due to the electrolyte coating. No hydrogen generation was reported for the control group either, since degradable polymeric composites were used. In study [34] hydrogen generated from uncoated magnesium alloy was gradually absorbed by the tissues, and no signs of gas generation were observed. However, the implants in the control group were not degraded.

### Fracture repair with zinc alloy materials (see Figs 3 and 4)

Only one included study [37] explored the repair of fractures with zinc alloy materials. A total of 24 animals were used. The animal species used was beagle dogs, aged 10–12 months, body weight 10–15 kg, and the follow-up time was 12 months. The fracture model was a mandible fracture model. The implants were four-hole bone plates (1 mm thick) and bone screws (2 mm diameter, 7 mm length) made of zinc alloy and PLLA.

The results were as follows. Compared with absorbable polymer materials, zinc alloy has better viability and osteogenesis in fracture healing and larger bone volume in the early stage of fracture healing and smaller volume in the middle stage of fracture healing. During the whole process of fracture healing, the maximum stress of the two groups both increased gradually, and there was no statistical difference between the groups. Remaining implant volume of the zinc alloy group gradually decreased as the implantation time prolonged. However, the remaining volume of implants in the control group was not reported.

### Results from assessing the risk of bias and quality of evidence

**Figure 5:**
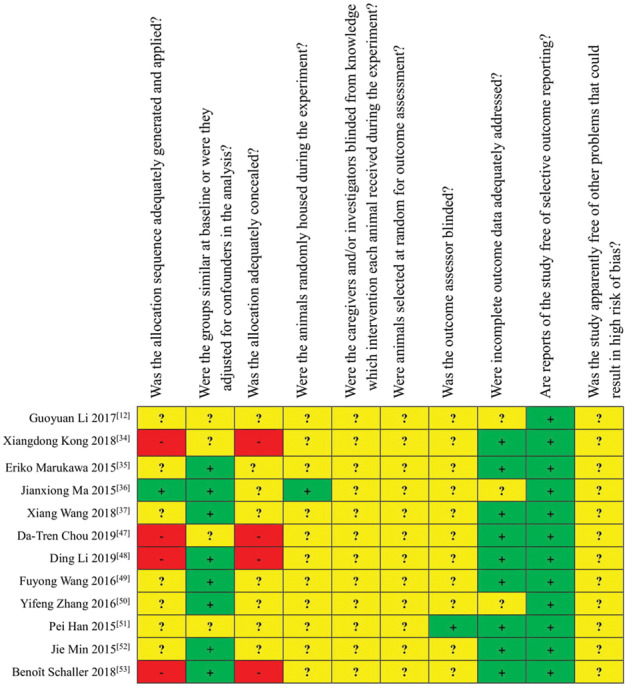
Results of the risk of bias assessment of the 12 studies included in this SR (the items were scored with ‘yes’, ‘no’, ‘unsure’ [54])

**Figure 6:**
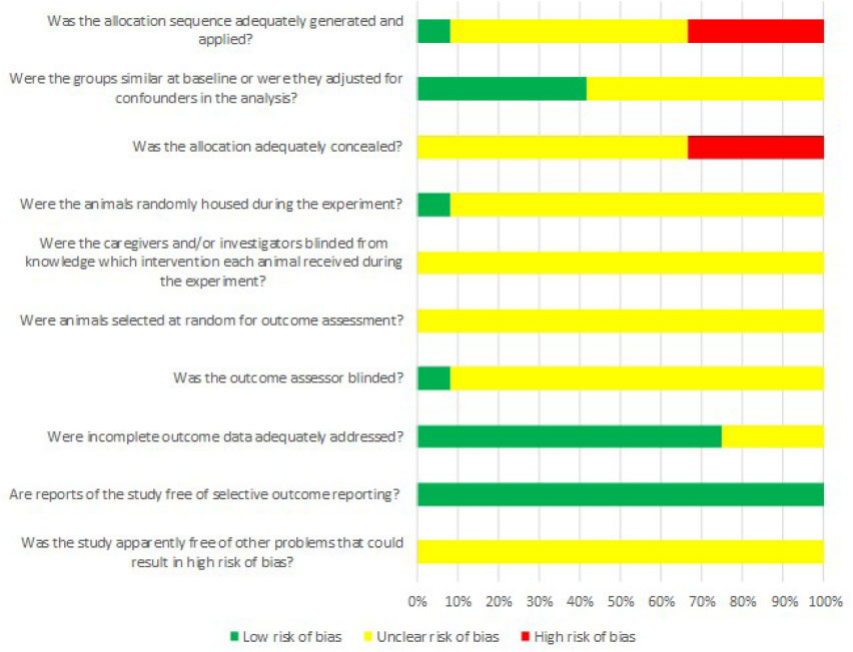
Risk of bias of each item of SYRCLE tool for overall included studies (each risk of bias item presented as percentages across all included studies, which indicated the proportion of different level risk of bias for each item [54])

The results of the bias risk assessment included in the study are shown in Figs 5 and 6. Among the 12 included animal studies, eight studies [12, 35–37, 49–52] were randomized controlled studies, with only one study [36] reported a specific randomized grouping method. The eight studies did not report whether sequence generation was concealed. Eight studies [35–37, 48–50, 52, 53] had balanced baseline characteristics. None of the studies reported whether caregivers and researchers were blinded. The methods of animal selection were not included during outcome assessment. Only one study [36] randomized placement of experimental animals. Only one study [51] reported blinding of outcome assessors. The experimental animals of nine studies [34, 35, 37, 47–49, 51–53] were included in the final analysis. Although no research protocol was available for any of the studies, all expected results were clearly reported.

The results from assessing the quality of evidence showed ‘very low’ quality in the eight outcome measures. The reasons for poor quality of evidence included lack of authenticity in original research, inconsistency of results, and difficulty in amalgamating and translating relevant data (Table 3).

**Table 3:** Summary of the confidence rating of outcomes (CERQual qualitative evidence profile table) [45, 46]

Outcome measures	Number of included studies	Aspect 1: methodological limitations	Aspect 2: correlation	Aspect 3: consistency of results	Aspect 4: adequacy of data	CERQual level
New bone formation	Eight [12, 34, 36, 49, 50–53]	Selection bias [49, 51, 52, 54, 55, 57, 58]; performance bias [12, 34, 36, 49, 51–53]; detection bias [12, 34, 36, 49, 51–53]; attrition bias [12, 36, 50]	The clinical translation is limited by the type of fractures, type and implant design of biodegradable metals, design and duration of implantation	Among eight included studies, only one study [36] showed the control group is superior to the biodegradable metals in terms of new bone formation, the rest studies showed opposite.	Only one study quantitatively measured new bone formation [53]	⊕⊝⊝⊝ very low
Fracture healing	Seven [35–37, 48, 49, 52, 53]	Selection bias [50–52, 54–56]; performance bias [35–37, 48, 49, 52, 53]; detection bias [35–37, 48, 49, 52, 53]; attrition bias [36]	The clinical translation is limited by the type of fractures, type and implant design of biodegradable metals, design and duration of implantation	Among seven included studies, the biodegradable metals group is superior to the control group in terms of fracture healing only for the following specific periods of the specific studies: T initial in the studies [37, 48], the terminal period in the studies [35, 52], the mid-term, long-term and terminal periods in the study [49]	Only one study quantitatively measured bone fracture healing [53]	⊕⊝⊝⊝ very low
Bone volume	Four [12, 36, 37, 50]	Selection bias [12, 37, 50]; performance bias [12, 36, 37, 50]; detection bias [12, 36, 37, 50]; attrition bias [12, 36, 50]	The clinical translation is limited by the type of fractures, type and implant design of biodegradable metals, design and duration of implantation	Among the four included studies, the bone volume for biodegradable metals group is smaller than that of the control group only for the following specific periods of the specific studies: the initial, mid- and long-term in the study [36], the mid-term in the study [37]	The bone volume was quantitatively measured in all studies. However, due to the great heterogeneity in the study design, animal species, age, fracture models, type and composition of biodegradable metals, implant design, duration of implantation, measurement time, measurement methods and criteria for outcome measures, it was impossible to conduct a meta-analysis on the data available in the included studies	⊕⊝⊝⊝ very low
Total bone callus volume	One [36]	Performance bias [36], detection bias [36], attrition bias [36]	The clinical translation is limited by the type of fractures, type and implant design of biodegradable metals, design and duration of implantation	The biodegradable metals group is better than the control group in terms of total bone callus volume only for mid- and long-term period in the study [36]	Only study [36] quantitatively measured total bone callus volume	⊕⊝⊝⊝ very low
Maximum stress	Five [51, 53, 56–58]	Selection bias [12, 37, 50, 51], performance bias [12, 36, 37, 50, 51], detection bias [12, 36, 37, 50, 51], attrition bias [12, 36, 50]	The clinical translation is limited by the type of fractures, type and implant design of biodegradable metals, design and duration of implantation	Among the five included studies, only the initial and terminal periods in the study [36] showed that the biodegradable metals group is worse than the control group in terms of maximum stress, the rest periods and studies showed the opposite	The maximum stress was quantitatively measured in all studies. However, due to the great heterogeneity in the study design, animal species, age, fracture models, type and composition of biodegradable metals, implant design, duration of implantation, measurement time, measurement methods and criteria for outcome measures, it was impossible to conduct a meta-analysis on the data available in the included studies	⊕⊝⊝⊝ very low
Volume of Remaining implant	Four [37, 47, 51, 53]	Selection bias [37, 47, 51, 53], performance bias [37, 47, 51, 53], detection bias [37, 47, 51, 53]	The clinical translation is limited by the type of fractures, type and implant design of biodegradable metals, design and duration of implantation	Among the four studies, only one study [53] shows there is no statistical difference between the biodegradable metals group and the control group. The other three studies did not even compare	The remaining implant volume was quantitatively measured in all studies. However, due to the great heterogeneity in the study design, animal species, age, fracture models, type and composition of biodegradable metals, implant design, duration of implantation, measurement time, measurement methods and criteria for outcome measures, it was impossible to conduct a meta-analysis on the data available in the included studies	⊕⊝⊝⊝ very low
Implant degradation	Five [34, 35, 47–49]	Selection bias [34, 35, 47–49], performance bias [34, 35, 47–49], detection bias [34, 35, 47–49]	The clinical translation is limited by the type of fractures, type and implant design of biodegradable metals, design and duration of implantation	Among the five included studies, only the initial period in the study [48] and the long-term period in the study [49] show that implant degradation of the biodegradable metal’s groupies more significant than that of the control group	Only study [47] quantitatively measured implant degradation	⊕⊝⊝⊝ very low
Hydrogen generation	Five [34, 47, 49, 52, 53]	Selection bias [34, 47, 49, 52, 53], performance bias [34, 47, 49, 52, 53], detection bias [34, 47, 49, 52, 53]	The clinical translation is limited by the type of fractures, type and implant design of biodegradable metals, design and duration of implantation	Among the five included studies, only the initial period in the study [47], and the initial and terminal period in the study [34] showed that, hydrogen generation of the biodegradable metals group is more significant than that of the control group.	Hydrogen generation was qualitatively measured in all studies	⊕⊝⊝⊝ very low

## Discussion

We systematically reviewed 12 animal studies that qualified for the inclusion criteria. However, due to the great heterogeneity in the study design, animal species, age, fracture models, type and composition of degradable metals, implant design, implantation time, measurement time, measurement methods and criteria for outcome measures, it was impossible to conduct a meta-analysis on the data available in the included studies. Hence, only a qualitative description and discussion are provided below.

### Biodegradable vs non-degradable metals

For bone fracture repair, in the included studies, compared to non-degradable metals, biodegradable metal implants yielded better results in promoting the formation of new bone in animal models of fractures, accelerating fracture healing, and contributing to the growth of bone and callus in the early stage of fracture healing. In the later stage of fracture healing, the fracture ends of the biodegradable metal group were neatly aligned and the fracture line disappeared. The new bone tissue also showed higher mechanical strength. However, the control group had poor fracture alignment and visible fracture line. Nevertheless, the results of study [36] showed that early fracture repair performance in the biodegradable metal group (reflected in new bone formation, bone volume and maximum stress) was worse than that of the non-degradable control group. This was probably due to the need for a strong and reliable initial fixation of orthopedic implants in the early stage of fracture healing. As a result, the iron intramedullary nails with a higher elastic modulus in the control group of study [36] exhibited better early fracture healing. Therefore, it is a key issue to ensure that the biodegradable metals have considerable strength to meet the mechanical support requirements at the early stage of fracture healing.

On the matter of degradation, different expectations were held in different studies. Some researchers expected that any degradation would be desirable, while others believed that only full degradation would meet their expectations. The results of studies [47, 48] showed that the biodegradable metal implants degraded at a faster rate throughout the fracture repair process, and achieved the expected degradation and fracture repair performance at the end of fracture healing. At the end of the follow-up process, the implants in study [47] degraded by 57%, while the implants of study [48] fully degraded. High-quality healing at the fracture lines was achieved for both studies. As a result, secondary surgery was avoided. However, the results of study [49] showed that the fracture healing performance of experimental animals was worse than those of the non-degradable metal group due to the rapid degradation of uncoated metal implants. In contrast, the non-degradable implants in this case provided strong mechanical support due to their resistance to degrading, and exhibited better facilitation effects for fracture healing. Therefore, the degradation rate of the biodegradable metals alone does not guarantee its application value in fracture repair. It is imperative for future research to consider the degradation rate of the biodegradable metals in the light of the fracture healing rate and aim for a more balanced outcome evaluation for fracture healing and repair.

### Biodegradable metals vs absorbable polymers

Compared with absorbable polymers, the biodegradable metal has better osteoblast activity, and higher quantity and quality of neovascularization and new bone trabeculae. The biodegradable metals benefit from better and more gradual degradation performance during the fracture healing process. The biodegradable metal groups showed enhanced fracture repair. However, the results of studies [34, 35] were contrary to these finding, in that the degradation of absorbable polymer biomaterial implants was significantly better than that of the biodegradable metal implants. These conclusions were critically inconsistent with the expectation that the degradable metal should degrade faster [61]. A potential cause may be the coating formed on the surface of the biodegradable metal, such as dicalcium phosphate dihydrate (DCPD) coating [34], and anodized layer [35], improving the corrosion resistance of metallic materials. In addition, studies [34, 35] did not report whether the degradation rate of biodegradable metals has any effect on the fracture healing and repair rates. Therefore, future research should further explore the specificity of degradation behavior in biodegradable metals for the benefit of fracture repair. At the same time, attention should be paid to the problem of aligning the degradation rate of biodegradable metals with the fracture healing rate. In addition, the biodegradable metals generated too much gas during the degradation process, which resulted in the formation of air cavities around the fracture sites and affected the healing effect, which could be one of the limitations of the available biodegradable metal implants [29].

In studies [34, 35, 53], the coating of biodegradable metals produced a better effect than fracture absorbable polymer biomaterials in promoting fracture healing. The inhibitory effect of the coating slowed down degradation of the biodegradable metals so that the hydrogen generated during the degradation process could be absorbed by the body in time without generating air cavities. Hence, surface coatings are considered effective means to reduce and control the corrosion behavior of biodegradable metals and improve biocompatibility [62]. However, there is still insufficient evidence to prove the safety and efficacy of coatings [63]. In addition, there are many coating methods and materials. The optimum thickness, uniformity, bonding force and durability of the coating have not been researched thoroughly. Therefore, the safety and efficacy of biodegradable metal coatings would be one of the key research topics for the future [63].

SRs were also conducted on biodegradable polymers such as PLA or polyglycolic acid (PGA) for bone regeneration in both animal and clinical studies [64], mandibular fixation in clinical studies [65] and fixation of metacarpal shaft fractures in clinical studies [23]. For bone regeneration, PLA was recommended to be modified by bioactive fillers such as tricalcium phosphate and hydroxyapatite [64]. For mandibular fixation, PLA implants did not provide conclusive data to support such applications in comparison with titanium plates [65]. For metacarpal shaft fractures’ fixation, PGA implants showed similar complication rates and biomechanical properties as compared with metallic implants [23].

### Sources of heterogeneity, internal authenticity and quality of evidence

Based on a rigorous SR, our research found that the current quality of evidence for the effect of biodegradable metals on fracture repair was low, reducing the reliability of the experimental results, and increasing the risks of translation with animal study results into the clinical practice. Possible reasons are explained as follows.

There were significant differences in the animal species, fracture models, measurement points, measurement methods, and criteria of outcome measures in the included studies. Consequently, the data acquired from the studies could not be meta-analyzed, which reduced the validity of the results. As an example, there was a total of 5 different animal species and 6 different fracture models in the 12 included studies. The primary problems with the outcome measures were variety and inconsistency. For any outcome measure, there would be different numbers of studies involved. For instance, eight studies [12, 34, 36, 49, 51–53] reported on new bone formation, seven studies [35–37, 48, 49, 52, 53] on fracture healing, five studies [34, 35, 47–49] on implant degradation, five studies [34, 47, 49, 52, 53] on hydrogen generation, four studies [12, 36, 37, 50] on bone volume, four studies [12, 36, 37, 50, 51] on maximum stress, one study [36] on total callus volume and four studies [37, 47, 51, 53] on remaining implant volume.

The outcome measures were captured by divergent approaches and methods. The same outcome measures were taken under different paradigms. For instance, new bone formation was quantitatively measured in study [53], whereas a qualitative approach was used in other studies [36, 51, 52]. The results of the same outcome measures came from different tools. To evaluate fracture healing, studies [48, 53] adopted histological staining, while study [37] resorted to Micro-CT for morphological observation. Different criteria were chosen for the same outcome measures. To interpret the new bone formation data, studies [12, 34, 36, 50, 51] adopted imaging or histological staining or other methods to observe the formation of new bone tissue, trabeculae and callus. However, the effect of new bone formation was judged in study [52] with imaging methods to assess the alignment status of fracture ends and whether the fracture line disappeared.

The study design of most of the included experiments was not rigorous and scientific. For example, the randomization process of the studies of 91.67% (11/12) studies was unspecified. None of the studies reported on sequence generation concealment. Baseline characteristics were uneven in 58.33% (7/12) of the studies. Consequently, the probability of selection bias was high. Compared with clinical trials, the sample size of most animal experiments was small. For instance, among the 12 studies included in this SR, 6 studies [34, 35, 37, 49, 52, 53] had fewer than 30 animal subjects. Some important differences in baseline characteristics will greatly affect the experimental results [54].

Most experiments lacked quality control measures to reduce measurement and implementation bias. For example, none of the studies reported whether caregivers/researchers or outcome assessors/raters were blinded. Although animal blindness is not required in animal experiments, most of the researchers are caregivers. Therefore, it is necessary to implement blindness during the intervention and outcome measurement stages to reduce implementation and measurement bias and increase the authenticity of the experimental results [66, 67]. For example, the measurement of new bone formation and fracture healing in the study of biodegradable metals for repair fractures mainly relies on researcher observation of the formation of bone trabeculae, callus and fracture line healing in or around the fracture line through imaging methods. If researchers have knowledge of the interventions in advance, they may be biased when evaluating the osteogenesis or fracture healing effect between the groups, affecting the authenticity of the results. In addition, the capture of outcome measures, especially those that depend on human judgment, it is imperative to implement an effective and scientific blind technique to avoid measurement bias on the results, but also have qualified technicians to ensure the inter/intra-rater consistency between different personnel on different animals, and accuracy of measurement calibration. All these potential biases have an impact on the results to varying degrees [68]. However, the 12 studies included in this SR did not report on the qualifications of the raters and the protocols and standards they follow for specific measurement processes.

Unbiased report of experimental data is needed. Although all the included studies clearly reported all expected results in their methods and results sections, we could not obtain their original research protocols, and ultimately judge whether they were implemented accordingly and all its results were reported in an unbiased manner. Selective reporting of animal experimental research results may lead to publication bias, which may affect the reliability of SR conclusions, and even lead to opposite conclusions [69].

### Publication bias

Experiments with positive results are usually more likely to be published than those with negative or null results [70, 71]. Prior studies [72] show that publication bias may be more severe in animal studies. Therefore, if SRs do not include unpublished studies, they are likely to produce overestimation of the effects of interventions. This study did not evaluate publication bias by statistical analysis. There was no safeguard that publication bias did not exist in this study. Therefore, in the field of experimental research, it is necessary to take measures to promote data sharing and encourage journals to publish studies with negative or neutral results to avoid the ‘file-drawer problem’ and reduce the impact of publication bias on their results [73].

### Strengths and limitations of this study

To the best of our knowledge, this is the first SR of animal studies to assess the performance of biodegradable metals in the treatment of orthopedic fractures. First, this review adopts the CERQual tool to evaluate the quality of evidence on the outcome measures. It provides an evidence-based assessment of the risk of translating preclinical results from animal studies to clinical trials. Second, the risk of bias in animal studies was assessed based on the internationally recognized SYRCLE tool. Third, the internal and external authenticity of the evidence is discussed in detail to objectively analyze the risk and feasibility of the translation of animal study results to clinical practice. However, there are two limitations for this SR. Searching only Chinese and English databases may result in certain language bias. Second, failure to search gray literature and conference abstracts may result in publication bias.

An updated literature search was conducted in August 2020. Three newly-published (between August 2019 and August 2020) studies [74–76] that met the inclusion criteria were identified. However, even with the inclusion of these three articles [74–76], the quality of evidence and the final conclusion of the current study remain the same.

### Prospects for future research

Through comprehensive analysis of the evidence in the included studies, including the risk of inherent bias, the quality of evidence, and outcome measures, we found that animal studies on biodegradable metals for repair of bone fractures has certain limitations. Therefore, except for exploring material-related issues such as the structure and performance of biodegradable metal materials, future research on biodegradable metals for repairing bone fractures would benefit from the quality of animal studies, which may further improve the translation of research results [77]. Specific improvement on animal studies include the following areas.

#### Selection of animal models

The current animal models for bone fractures are limited to non-primate animals such as rats, rabbits and pigs, and they differ greatly from the human body in terms of anatomical structure, biological characteristics and disease mechanisms [78]. It is recommended to standardize animal models for research on biodegradable metal materials for fracture repair in the future. The establishment of animal models should accommodate the differences between the bone physiological structure, structural clustering, bone metabolism and healing cycle of experimental animals and humans to identify appropriate model animals.

#### Fracture models

Different fracture models were used in the 12 studies including femoral fractures [12, 36, 47, 48, 50], femoral condyle fractures [34, 51], tibial fractures [35, 49], superior orbital margin and zygomatic fractures [53], rib fractures [52] and mandibular fractures [37]. Therefore, standardization of fracture modeling is recommended. The biomechanical environment of the intended use site of clinically implants should be considered, e.g. lining up the strength of the initial fixation needed at the fracture site and that of the implant. In the end, a representative animal model of fractures would be identified to evaluate the potential for clinical translation of biodegradable metals.

#### Study design (randomization and blindness)

One in three of the included studies were not randomized controlled study, and the majority of the 8 randomized studies did not report-specific randomization methods [12, 35, 37, 49–52] and sequence generation concealment methods [12, 34–37, 47–53]. Most of the included studies did not report blinding of caregivers/researchers [12, 34–37, 47–53] or outcome assessors [12, 34–37, 48–53]. Previous studies [79–82] show that randomized and concealed sequence generation and blindness are important measures to reduce the risk of inherent bias in animal experiments. Strict control of various risks of bias will help reduce risk of clinical translation from animal study results. Therefore, when designing animal studies in the future, rigorous design concepts such as randomness and blindness should be followed to reduce selection bias and improve the quality of animal studies.

#### Experiment implementation and quality control

The 12 studies included in this SR did not report on the qualifications of the outcome assessors and the assessment protocols and processes, nor did they mention the use of third-party evaluation. The sample size varied greatly. For example, study [12] included 60 animals in the trials, while study [53] used only 4 animals. Therefore, future research would benefit from scientifically rigorous methods to estimate the viability of sample size [83], and comprehensively report the experimental implementation details. This practice would improve the validity and reliability of animal study results. Randomization and blindness would be applied in the experimental design and implementation to ensure the authenticity of experimental results [84].

#### Selection, calculation and assessment of outcome measures

In the current research, there are no consistent standards indicating which outcome measures reflect the efficacy and safety of biodegradable metals for repair bone fractures, leading to large divergences in the outcome measures used in the included studies. The selection of improper outcome measures may lead to a huge waste of experiment animals and incorrect conclusions [85]. The large differences in the measurement and assessment methods for the same outcome measure in the included studies lead to increased heterogeneity and made it impossible to integrate and analyze data across different studies. Therefore, it is advisable to standardize the calculation and assessment of the outcome measures and adopt uniform standards to specify outcome measures that can best reflect the safety and effectiveness of biodegradable metals for fracture repair.

#### Reporting raw data

Government agencies and trade associations should encourage prospective registration of animal studies to obtain raw data [86]. It is very necessary for future animal study to share raw data as online appendices [86], enhancing research transparency and promoting quality of animal studies.

## Conclusions

Compared to controls of traditional non-degradable metals or resorbable polymers, biodegradable metals may have shown better outcomes in terms of fracture healing and degradation in animal models. However, such optimal results were not consistent, because there were studies also suggested that biodegradable metals did not demonstrate better performance for bone repair in animal models as compared to controls. Furthermore, the fast degradation rate of biodegradable metals may further impede fracture healing *in vivo*. The performance of biodegradable metals for bone fracture repair is uncertain because there are many issues of the included studies in terms of study design, outcome measurements as well as quality of evidence. Based on this study, reliable evidence from animal studies are needed to support future clinical translation of biodegradable metals for bone fracture repair. In order to better evaluate performance of bone fracture repair as well as to reduce the risks for the clinical translation of biodegradable metals, standardized study design and practice is a must for future animal studies.

## Supplementary data

Supplementary data are available at *REGBIO* online.

*Conflict of interest statement.* None declared. 

## Funding

We acknowledge the financial support from the National Natural Science Foundation of China (81873184).

## Supplementary Material

rbaa047_Supplementary_Data
