# Supplementary material for: Biodegradable metals for bone fracture repair in animal models: a systematic review
Source: Regen Biomater. 2020 Dec 3;8(1):rbaa047. doi: 10.1093/rb/rbaa047 (PMC7947587; doi:10.1093/rb/rbaa047)
Supplement: rbaa047_Supplementary_Data [file rbaa047_supplementary_data.zip › suppl_data/Supplementary material .doc]

**Supplemental data:**

**Chinese and English search strategies**

Comments:

In order for non-Chinese readers to understand the Chinese search strategy of this article, we translated the Chinese search terms in the search formula.

**Pubmed:2019.08**

1. (biodegradable metal OR degradable metal OR biodegradable alloy OR degradable alloy OR absorbable metal) OR ((biodegradable implants OR biodegradable fixation OR absorbable implants OR bioabsorbable implants OR biodegrading implants) AND (metal OR alloy OR magnesium OR Mg OR zinc OR Zn OR Iron OR Fe)) (4746)
2. "Fractures, Bone"[Mesh] OR fracture*[tiab] (286942)
3. Bone defect*[tiab] (12173)
4. "Fracture Healing"[Mesh] OR "Fracture Healing"[tiab] OR "fracture fixation"[Mesh] OR ("fracture"[tiab] AND "fixation"[tiab]) OR "fracture fixation"[tiab] (78787)
5. Bone repair*[tiab] OR bone heal[tiab] OR bone healed[tiab] OR bone heals[tiab] OR bone healing[tiab] (20864)
6. Bone fill*[tiab] (1070)
7. "Bone Screws"[Mesh] OR "Bone Screws"[tiab] OR "Bone Plates"[Mesh] OR "Bone Plates"[tiab] OR "Bone Nails"[Mesh] OR "Bone Nails"[tiab] OR intramedullary nail*[tiab] OR "pins"[tiab] (50884))

8. #2 OR #3 OR #4 OR #5 OR #6 OR #7 (332180) (491704)

9. Search filter for animal studies ^[1]^ (6834740) 10. #1 AND #8 AND #9 (322)

**Embase(Ovid):1980-2019.08**

1. (biodegradable metal or degradable metal or biodegradable adj3 alloy or degradable adj3 alloy or absorbable metal or ((biodegradable implants or biodegradable fixation or absorbable implants or bioabsorbable implants or biodegrading implants) and (metal or alloy or magnesium or Mg or zinc or Zn or Iron or Fe))).mp. (478)
2. exp fracture/ (261118)
3. exp bone defect/ (12188)
4. exp fracture healing/ or exp fracture fixation/ (105512)
5. exp bone filler/ (392)
6. exp bone screws/ or exp bone plates/ or exp bone nails/ (51306)
7. (fractur$ or (bone adj3 defect$)).mp. (369797)
8. (fracture healing or fracture fixation or (bone adj3 fill$) or bone screw$ or bone plate$ or bone nail$ or pins or (bone adj3 repair$) or (bone adj3 heal$)).mp. (111228)

9.	or/2-8 (428562)

10. Search filter for animal studies ^[2]^ (6287361) 11. And/1,9,10 (53)

[1] Hooijmans CR, Tillema A, Leenaars M, Ritskes-Hoitinga M (2010) Enhancing search efficiency by means of a search filter for finding all studies on animal experimentation in PubMed. Lab Anim 44: 170–175.
[2] de Vries RB, Hooijmans CR, Tillema A, Leenaars M, Ritskes-Hoitinga M (2011) A search filter for increasing the retrieval of animal studies in Embase. Lab Anim 45: 268–270.

**Cochrane library 2019.08**

1. ((biodegradable metal OR degradable metal OR biodegradable near/3 alloy OR degradable alloy OR absorbable metal) OR ((biodegradable implants OR biodegradable fixation OR absorbable implants OR bioabsorbable implants OR biodegrading implants) AND (metal OR alloy OR magnesium OR Mg OR zinc OR Zn OR Iron OR Fe))):ti,ab,kw (486)
2. MeSH descriptor: [Fractures, Bone] explode all trees (5447)
3. MeSH descriptor: [Fracture Healing] explode all trees (505)
4. MeSH descriptor: [Fracture Fixation] explode all trees (1604)
5. (fracture* or (bone near/3 defect*) or (Fracture Healing) or (Fracture Fixation) or (bone near/3 repair*) or (bone near/3 fill*) or (bone near/3 heal*)):ti,ab,kw (22889)
6. MeSH descriptor: [Bone Screws] explode all trees (754)
7. MeSH descriptor: [Bone Plates] explode all trees (555)
8. MeSH descriptor: [Bone Nails] explode all trees (413)
9. (Bone Screw* or Bone Plate* or Bone Nail* or pins or intramedullary nail*):ti,ab,kw (7647) 10. #2 or #3 or #4 or #5 or #6 or #7 or #8 or #9	(27637)

11. (animal* or animal experiment or animal stud* or animal trail or nonhuman or rat* or mice or pig* or swine* or dog* or sheep* or goat* horse* or oranguta* or monkey) (520806)

12. #1 and #10 and #11 (34)

**Web of science** WOS, BIOSIS, KJD, RSCI, SCIELO Time span = all years, search language = automatic

2019.08

1. TS=(biodegradable metal OR degradable metal OR Biodegradable alloy OR degradable alloy OR absorbable metal) OR (TS=(biodegradable implants OR Biodegradable fixation OR absorbable implants OR bioabsorbable implants OR biodegrading implants) AND TS=(metal OR alloy OR magnesium OR magnesia OR zinc OR Zn OR Iron OR Fe)) (9589)
2. TS=(fracture* OR bone defect*) OR TS=(Fracture Healing or Fracture Fixation or bone repair* OR bone fill* OR bone heal OR bone healed OR bone heals OR bone healing) OR TS=(Bone Screw* or Bone Plate* or Bone Nail* or pins or intramedullary nail*) (1038752)
3. TS=(animal* or animal experiment or animal stud* or animal trail or nonhuman or rat* or mice or pig* or swine* or dog* or sheep* or goat* or horse* or oranguta* or monkey) (22635815)

4. #1 AND #2 AND #3 (1088)

**CNKI/专业检索:2019.08 CNKI / Professional Search: 2019.08**

(SU % '可降解金属' OR SU % '可降解合金' OR SU % '镁合金' OR SU % '锌合金' OR SU % '铁合金' OR SU = '生物可降解固定物' OR SU % '可吸收植入物' OR SU = '可降解植入物') AND (SU = '骨折' OR SU = '骨缺损' OR SU = '骨折愈合' OR SU = '骨折固定' OR SU = '骨修复' OR SU = '骨填充' OR SU = '骨钉' OR SU = '骨板' OR SU = '骨针') AND (FT = '动物实验' OR FT = ' 动物' OR FT = '鼠' OR FT = '羊' OR FT = '猪' OR FT = '犬' OR FT = '牛' OR FT = '猩猩' OR FT = '马' OR FT = '猴') (131)

(SU % ' Degradable metal ' OR SU % ' Degradable alloy ' OR SU % ' Magnesium alloy ' OR SU % ' Zinc alloy ' OR SU % ' Iron alloy ' OR SU = ' Biodegradable fixation ' OR SU % ' Absorbable implant ' OR SU = ' Degradable implant ') AND (SU = ' fracture ' OR SU = ' bone defect' OR SU = ' fracture healing ' OR SU = ' fracture fixation' OR SU = 'Bone Repair' ORSU = 'Bone Filling'ORSU =' Bone Nail'ORSU = 'Bone Plate'ORSU =' Bone Needle ') AND (FT =' Animal Experiment ' ORFT = 'Animal'ORFT =' Rat'ORFT = 'Sheep'ORFT =' Pig'ORFT = 'Dog'ORFT =' Bull'ORFT = 'Orangutan'ORFT =' Horse'ORFT = 'Monkey') (131)

**万方/专业检索:2019.08 WanFang datebase / Professional Search: 2019.08**

(主题: (生物可降解金属 OR 可降解金属 OR 生物可降解合金 OR 可降解合金 OR 可降解镁合金 OR 可降解锌合金 OR 可降解铁合金) OR 主题:(生物可降解植入物 OR 生物可降解固定物 OR 可吸收植入物 OR 生物可吸收植入物 OR 可降解植入物)) AND 主题: (" 骨折" OR "骨缺损" OR "骨折愈合" OR "骨折固定" OR "骨修复" OR "骨填充" OR "骨钉" OR "骨板" OR "骨针") AND 主题: ("动物" OR "动物实验" OR "鼠" OR "羊" OR "猪" OR "犬" OR "牛" OR "猩猩" OR "马" OR "猴") (474)

(Subject: (Biodegradable metal OR Degradable metal OR Biodegradable alloy OR Degradable alloy OR Degradable magnesium alloy OR Degradable zinc alloy OR Degradable iron alloy) OR Subject: (Biodegradable implant OR Biodegradable Fixture OR Resorbable implant OR Bioresorbable implant OR Degradable implant) AND Subject: ("fracture" OR "bone defect" OR "fracture healing" OR "fracture fixation" OR "bone repair" OR "bone filling" OR "bone nail" OR "bone plate" OR "bone needle") AND Subject: ("Animal" OR "Animal Experiment" "OR" Rat "OR" Sheep "OR" Pig "OR" Dog "OR" Cow "OR" Orangutan "OR" Horse "OR" Monkey ") (474)

**维普/专业检索:2019.08 VIP datebase / Professional Search: 2019.08**

M=(生物可降解金属 OR 可降解金属 OR 生物可降解合金 OR 可降解合金 OR 可降解镁合金 OR 可降解锌合金 OR 可降解铁合金 OR 生物可降解植入物 OR 生物可降解固定物 OR 可吸收植入物 OR 生物可吸收植入物) AND M=(骨折 OR 骨缺损 OR 骨折愈合OR 骨折固定 OR 骨修复 OR 骨填充 OR 骨钉 OR 骨板 OR 骨针) AND U=(动物 OR 动物实验 OR 鼠 OR 羊 OR 猪 OR 犬 OR 牛 OR 猩猩 OR 马 OR 猴) (7)

M = (biodegradable metal OR degradable metal OR biodegradable alloy OR degradable alloy OR degradable magnesium alloy OR degradable zinc alloy OR degradable iron alloy OR biodegradable implant OR biodegradable fixation OR resorbable implant OR bioresorbable implant) AND M = (fracture OR bone defect OR fracture healing OR fracture fixation OR bone repair OR bone filling OR bone nail OR bone plate OR bone needle) AND U = (Animal OR Animal Experiment OR Rat OR Sheep OR Pig OR Dog OR Cattle Orangutan OR Horse OR Monkey) (7)

**CBM/高级检索:2019.08 CBM / Advanced Search: 2019.08**

( "生物可降解金属"[常用字段:智能] OR "可降解金属"[常用字段:智能] OR "生物可降解合金"[常用字段:智能] OR "可降解合金"[常用字段:智能] OR "镁合金"[常用字段:智能] OR "锌合金"[常用字段:智能] OR "铁合金"[常用字段:智能] OR "生物可降解植入物"[常用字段:智能] OR "生物可降解固定物"[常用字段:智能] OR "可吸收植入物"[常用字段:智能] OR "生物可吸收植入物"[常用字段:智能]) AND( "骨折"[常用字段:智能] OR "骨缺损"[常用字段:智能] OR " 骨折愈合"[常用字段:智能] OR " 骨折固定"[常用字段:智能] OR "骨修复"[常用字段:智能] OR "骨填充 OR 骨钉"[常用字段:智能] OR "骨板"[常用字段:智能] OR "骨针"[常用字段:智能])

AND( "动物"[常用字段:智能] OR "动物实验"[常用字段:智能] OR "鼠"[常用字段:智能] OR "

羊"[常用字段:智能] OR "猪"[常用字段:智能] OR "犬 OR 牛"[常用字段:智能] OR "猩猩"[常

用字段:智能] OR "马"[常用字段:智能] OR "猴"[常用字段:智能]) (13)

( "Biodegradable metal" [common field: smart] OR "degradable metal" [common field: smart] OR "biodegradable alloy" [common field: smart] OR "degradable alloy" [Common Field: Smart] OR "Magnesium Alloy" [Common Field: Smart] OR "Zinc Alloy" [Common Field: Smart] OR "Iron Alloy" [Common Field: Smart] OR "Biodegradable implant" [Common field: Smart] OR "Biodegradable fixation" [Common field: Smart] OR "Absorbable implant" [Common Field: Smart] OR "Bioabsorbable Implant" [Common Field: Smart]) AND("Fracture" [common field: intelligent] OR "bone defect" [common field: smart] OR "fracture healing" [common field: smart] OR "fracture fixation" [common field: smart] OR "bone repair" [Common Field: Smart] OR "Bone Fill OR Bone Nail" [Common Field: Smart] OR "Bone Plate" [Common Field: Smart] OR "Bone Needle" [Common Field: Smart]) AND ("Animal" [Common Field: Smart] OR "Animal Experiment" [Common Field: Smart] OR "Rat" [Common Field: Smart] OR "Sheep" [Common Field: Smart] OR "Pig" [Common Field: Smart] OR "Dog OR Cattle" [Common Field: Smart] OR "Gorilla" [Common Field: Smart] OR "horse" [Common Field: Smart] OR "Monkey" [Common Field: Smart]) (13)
