# Supplementary material for: Biodegradable metals for bone fracture repair in animal models: a systematic review
Source: Regen Biomater. 2020 Dec 3;8(1):rbaa047. doi: 10.1093/rb/rbaa047 (PMC7947587; doi:10.1093/rb/rbaa047)
Supplement: rbaa047_Supplementary_Data [file rbaa047_supplementary_data.zip › suppl_data/Reply to Reviewers Comments.docx]

Response to reviewers

**Reviewer #1:**

**Question 1: For the content of the research work, since elements Fe is mainly distributed in red blood cell, and the application of Fe within is rarely reported, although papers less than 10 were published, they were believed not the major research direction. Additionally, since the research work on Zinc for orthopedic application is just starting, and in this paper only one literature had been selected, it is better to delete the related content, and focus on Mg based biodegradable metals only. Accordingly, it is better to modify the title of this paper, to "Mg-based biodegradable metals".**

*The objective of this research is to conduct a systematic review on the animal studies of biodegradable metals for bone fracture repair. As a result, the search is designed to include studies of all biodegradable metals that meet the inclusion criteria. Furthermore, systematic reviews as an evidence-based research method, can scientifically evaluate the strength and weakness of published and completed studies. For example, although only one article related to Zinc and its alloy was included, the data can both reveal the current issues and provide evidence or references of this kind of biodegradable metal for bone fracture repair in animal models.*

*Based on the study design, Fe and Zn based biodegradable metals-related studies should all be searched and included/excluded per the inclusion and exclusion criteria.*

*Following the suggestions of both reviewer #1 and #3, we revised the title to “Biodegradable Metals for Bone Fracture Repair in Animal Models: A Systematic Review” in order to better represent the content of this manuscript.*

"Biodegradable Metals for Bone Fracture Repair in Animal Models: A Systematic Review"

**Question 2: By the criteria using in this study, some important references (ESI highly cited papers) in the field of biomedical Mg alloys had been excluded, please explain it. For instance, Biomaterials 26 (2005) 3557–3563; Biomaterials 29 (2008) 1329-1344; Nature Materials, 2009, 8 (11): 887-891; www.pnas.org/cgi/doi/10.1073/pnas.1518238113.**

*Authors investigated the references that the reviewer mentioned in the comment. These four articles all did not meet the inclusion criteria of the current study design. In order to address the concerns that the reviewer has raised, the following content and references were added to the end of Section 3.2 (page 24, line 21-22).*

…Based on the inclusion criteria, some of the highly-cited research articles on biodegradable metals were excluded, due to non-fracture models [59-62] and self-control [60] of those animal studies.

[59] Witte F, Kaese V, Haferkamp H et al. In vivo corrosion of four magnesium alloys and the associated bone response. Biomaterials 2005; 26:3557-3563.

[60] Li Z, Gu X, Lou S, Zheng Y. The development of binary Mg-Ca alloys for use as biodegradable materials within bone. Biomaterials 2008; 29:1329-1344.

[61] Zberg B, Uggowitzer PJ, Löffler JF. MgZnCa glasses without clinically observable hydrogen evolution for biodegradable implants. Nat Mater 2009; 8:887-891.

[62] Lee JW, Han HS, Han KJ et al. Long-term clinical study and multiscale analysis of in vivo biodegradation mechanism of Mg alloy. Proc Natl Acad Sci U S A 2016; 113:716-721.

**Question 3: For the same methodology, had the authors study other biomaterials for bone fracture repair? How is this method applicable? Alternatively, since PLA is used in the present as control group, if using this method on the PLA for bone fracture repair, how is the reliable evidence from animal studies? For the discussion part, please consider to add some comments on this point.**

*The same approach (i.e., systematic review) has been used to study other biomaterials for both preclinical and clinical settings. As suggested by the reviewer, authors conducted a search on biodegradable polymers such as polylactic acid (PLA) or polyglycolic acid (PGA), which identified that PLA or PGA had been studied with systematic reviews for bone regeneration in both animal and clinical studies, mandibular fixation in clinical studies and fixation of metacarpal shaft fractures in clinical studies. The following content as well as three references were added to the last paragraph in Section 4.2 (page 49, line 4-11).*

Systematic reviews were also conducted on biodegradable polymers such as polylactic acid (PLA) or polyglycolic acid (PGA) for bone regeneration in both animal and clinical studies [66], mandibular fixation in clinical studies [67] and fixation of metacarpal shaft fractures in clinical studies [68]. For bone regeneration, PLA was recommended to be modified by bioactive fillers such as tricalcium phosphate and hydroxyapatite [66]. For mandibular fixation, PLA implants did not provide conclusive data to support such applications in comparison with titanium plates [67]. For metacarpal shaft fractures’ fixation, PGA implants showed similar complication rates and biomechanical properties as compared with metallic implants [68].

[66] Rajendran T, Venugopalan S. Role of Polylactic Acid in Bone Regeneration-A Systematic Review. Journal of Pharmaceutical Sciences and Research 2015; 7:960.

[67] Agarwal S, Gupta A, Grevious M et al. Use of resorbable implants for mandibular fixation: a systematic review. Journal of Craniofacial Surgery 2009, 20:331-339.

[68] Hazan J, Azzi A J, Thibaudeau S. Surgical fixation of metacarpal shaft fractures using absorbable implants: a systematic review of the literature. Hand 2019, 14: 19-26.

**Reviewer #2:**

**Question 1: The whole article needs to be polished and grammatical errors corrected by the native English author.**

*The English of this manuscript has been completely polished and improved. The revised manuscript represented the best English presentation of authors.*

**Reviewer #3:**

**Question 1: The title of the manuscript could be concise and precise. The reviewer recommends a title of “Biodegradable Metals for Bone Fracture Repair in Animal Models: A Systematic Review”.**

*The title of this manuscript has been changed to “Biodegradable Metals for Bone Fracture Repair in Animal Models: A Systematic Review”.*

"Biodegradable Metals for Bone Fracture Repair in Animal Models: A Systematic Review"

**Question 2: This manuscript is a bit long. The authors are encouraged either to move some main content to supplemental data or remove some detailed description. For example, Table 1 and its related information could be moved to supplemental data. In addition, information related to Figure 2 (i.e., contents in the second paragraph in Section 3.1) could be removed.**

*The content of this manuscript was reduced by moving some content to supplemental data and removing some detailed description. Specifically, Table 1 and its related information was moved to supplemental data. In addition, information related to Figure 2 was removed.*

"~~Table 1 showed the search strategy for PubMed.~~ See supplemental data for Chinese and English search strategies. "

"~~31 in vitro study articles, 9 articles using resorbable biomaterials as interventions, 99 articles that used either biodegradable metals or empty control as control groups, 6 article that did not use the designated outcomes, 5 proceeding abstracts that could not get full texts, 5 duplicates, and 23 articles on biodegradable metals for bone defect filling were excluded from the above 190 articles.~~ 12 animal studies on biodegradable metals for bone fracture repair were finally included ~~from the above 190 articles~~, including 8 English [47-52, 57,58] and 4 Chinese articles [53-56]. The screening and selection processes are shown in Figure 2. Animal studies on biodegradable metals for bone fracture repair were conducted by several groups in recent years. The 12 included studies from China, America, Japan and Switzerland were published between 2015 and 2019. "

**Question 3: The literature search was conducted in August 2019. Although it takes time to conduct evidence-based research, and the reviewer still wonders if newly-published articles in the past year related to this study may impact the results or conclusion of this study. The authors are encouraged to comments and/or add new information, if necessary and available.**

*Systematic review is typically good for two years, beyond which an updated search will be needed. The current study conducted the search in August 2019. In order to address the reviewer’s question, an updated search was performed and three new references which met the inclusion criteria were identified. However, the conclusion of this current study did not change. As a result, the following information as well as three new references were added as the second paragraph in Section 4.5 (page 51, line 27-30).*

An updated literature search was conducted in August 2020. Three newly-published (between August 2019 and August 2020) studies [77-79] that met the inclusion criteria were identified. However, even with the inclusion of these three articles [77-79], the quality of evidence and the final conclusion of the current study remain the same.

[77] Byun SH, Lim HK, Cheon KH et al. Biodegradable magnesium alloy (WE43) in bone-fixation plate and screw. J Biomed Mater Res B Appl Biomater 2020; 108:2505-2512.

[78] Wang Z, Wang X, Tian Y et al. Degradation and osteogenic induction of a SrHPO4-coated Mg-Nd-Zn-Zr alloy intramedullary nail in a rat femoral shaft fracture model. Biomaterials 2020; 247:119962.

[79] Imwinkelried T, Beck S, Schaller B. Pre-clinical testing of human size magnesium implants in miniature pigs: Implant degradation and bone fracture healing at multiple implantation sites. Mater Sci Eng C Mater Biol Appl 2020; 108:110389.

**Question 4: Authors need to revise the Abstract. Specifically, the last sentence in the Abstract, “Based on current findings, there is no reliable evidence of bone fracture repair in animal models to ensure future clinical translation of biodegradable metals” can be revised as “Evidence-based biomaterials research could help to identify reliable scientific evidence to ensure future clinical translation of biodegradable metals for bone fracture repair.”**

*The last sentence in the Abstract was revised to “Evidence-based biomaterials research could help to both identify reliable scientific evidence and ensure future clinical translation of biodegradable metals for bone fracture repair”.*

**…**Evidence-based biomaterials research could help to both identify reliable scientific evidence and ensure future clinical translation of biodegradable metals for bone fracture repair.

**Question 5: Authors also need to revise the Conclusion. Specifically, “Based on this study, there is no reliable evidence from animal studies to support future clinical translation of biodegradable metals for bone fracture repair.” can be revised as “Based on this study, reliable evidence from animal studies are needed to support future clinical translation of biodegradable metals for bone fracture repair.”**

*The Conclusion was revised as suggested. Specifically, “Based on this study, there is no reliable evidence from animal studies to support future clinical translation of biodegradable metals for bone fracture repair.” was revised to “Based on this study, reliable evidence from animal studies are needed to support future clinical translation of biodegradable metals for bone fracture repair.”*

Based on this study, reliable evidence from animal studies are needed to support future clinical translation of biodegradable metals for bone fracture repair.
